# Incidence of acute transfusion reactions and associated factors among adult blood-transfused patients at Jimma University Medical Center, southwest Ethiopia: A cross-sectional study

**DOI:** 10.1097/MD.0000000000039137

**Published:** 2024-08-09

**Authors:** Edosa Tadasa, Wondimagegn Adissu, Misgana Bekele, Gebeyaw Arega, Lealem Gedefaw

**Affiliations:** a School of Medical Laboratory Sciences, Faculty of Health Sciences, Institute of Health, Jimma University, Jimma, Ethiopia; b Clinical Trial Unit, Jimma University, Jimma, Ethiopia.

**Keywords:** acute transfusion reaction, blood transfusion, hemovigilance, southwest Ethiopia

## Abstract

Acute transfusion reaction is mainly related to the infusion of blood or blood products resulting at any time within a day of the intervention. It ranges from a non‐specific febrile episode to a life-threatening intravascular hemolysis. The severity of the reaction and the degree of morbidity are usually related to the degree of ABO incompatibility and the volume of blood transfused. Therefore, this study aimed to determine the incidence of acute transfusion reactions and its associated factors in Jimma University Medical Center, southwest Ethiopia. Institution-based cross-sectional study was conducted from 1 October to December 30, 2020. A total of 384 transfused patients were followed in this study. Socio-demographic and clinical data were collected through a structured questionnaire. Baseline measurement and 24-hour periodic vital signs monitoring were conducted after each transfusion. Four milliliters of venous blood were drawn after transfusion intervention from each distrusted patient for complete blood count, blood group phenotype, direct antihuman globulin test (DAT), and crossmatching. Data were entered into Epi data version 3.1 and analyzed using Statistical Package for Social Science software (SPSS) version 20. Descriptive statistics, and bivariable and multivariable logistic regression were employed to test the association between independent and dependent variables. A *P* value ≤ .05 was considered to indicate statistical significance. Acute transfusion reactions were diagnosed in 5.7% of patients, with most of these reactions were febrile nonhemolytic reactions (63.6%) followed by allergic (36.4%) reactions with mild clinical manifestations (27.3%). Transfusion history, transfused blood that was kept for more than 13 days, abortion history, and number of transfused units (≥3 units of blood/blood component) have 3.3, 3.85, 4.2, and 3.9 times greater odds, respectively, besides their significant association with the incidence of acute transfusion reactions. Patients with a history of previous transfusion, abortion, multi-unit transfusion, and patients transfused with blood stored for ≥14 days should be closely monitored. Starting a hemovigilance system of monitoring, collecting, and evaluating data on adverse effects of blood transfusion locally and nationally will decrease the occurrence of acute transfusion reactions.

## 1. Introduction

At large, blood component transfusions are an effective means of correcting a temporary deficiency of red blood cells (RBCs), platelets (PLTs), and coagulation factors.^[1]^ The main objectives of blood transfusion are to treat chronic anemia, coagulopathy, life-threatening bleeding disorders, and ineffective erythropoiesis.^[2,3]^ In certain clinical situations or to recover from serious diseases, blood transfusion can be the only way to save life. However, the transfusion process involves clinical risks with the potential occurrence of acute or delayed transfusion reactions.^[4]^

Acute transfusion reaction (ATR) occurs within a day of the primary blood infusion practice which includes febrile nonhemolytic transfusion reaction (FNHTR), transfusion-related acute lung injury (TRALI), transfusion-associated circulatory overload (TACO), isolated hypotension, bacterial contamination, allergic (minor, moderate or severe) reactions and anaphylactic reactions, and acute hemolytic reaction (AHR).^[5,6]^ ATRs rates vary from 0.5% to 3% of every transfusion episode during or within a few hours of infusions of blood or blood products.^[7]^ Data from serious hazards of transfusion (SHOT) annual reports indicated a scarce number of mild FNHTR, while an incidence of more clinically serious ATR of around 14/100,000 components transfusions, ranging from 11/100,000 for packed red cells (PRBCs) to 29/100,000 for platelet concentrates (CPLT).^[8]^

According to Samukange et al, the global adverse reactions (Ars) or adverse events (AEs) on VigiBase, a global database for medicine safety, in 2021 were ARs or AEs reported as 10.9% for blood and blood components, 88.4% for plasma derivatives, and 0.7% for recombinant blood products worldwide. According to the research, the most common ARs for blood and blood components are allergic and anaphylactic reactions (25.4%), followed by febrile nonhemolytic responses (15.9%), TACO (12.5%), and hemolytic transfusion reactions (11.8%).^[9]^ With limited data on ATRs in Africa and Ethiopia; Hume et al based in Uganda reported 11 acute reactions occurrences from 337 whole blood-platelet units (WB-Pus), involving 13 units of WB-PUs, resulting in a transfusion reaction rate of 7.3% (CI 3.7–12.7%) per transfusion episode, the majority of reported reactions were febrile nonhemolytic transfusion reaction, allergic transfusion reaction, and transfusion-associated dyspnea.^[10]^ In northwest Ethiopia, ATRs were observed in 5.2% of patients, out of which 65% were due to an allergic reaction, 30% due to FNHTRs, and 5% attributed to alloimmunization.^[11]^

AHR is usually the result of an error and is estimated to occur in approximately 1 in every 30 to 70,000 PRBC transfusions and associated mortalities are estimated to occur in approximately 1:800,000 of transfusions.^[12,13]^ As one of the common types of ATRs, FNHTR mainly forced clinical settings of universal leuko-reduction of blood supplies before actual transfusion. The frequency of FNHTR ranges from 0.15% to 0.19% for red cells and 0.11% to 0.15% for platelet transfusions. When non-leuko-reduced (leuko-depleted) blood products are routinely administered, the frequency of FNHTRs increased to 0.33% to 0.37% for red cells and 0.55% to 2.18% for platelet transfusions.^[14–16]^

Along with FNHTRs, mild allergic reactions are arguably the most common type of transfusion reaction.^[16]^ Allergic reactions are common, with an overall incidence of 0.4% to 3% of transfusions.^[17]^ Most reactions involve urticarial alone. Anaphylactic reactions occur rarely ranging from 1:20,000 to 1:50,000 transfusions episodes.^[17,18]^

The estimated incidence of TRALI on the other hand is between 0.04% and 0.1% of all transfusions.^[19,20]^ It is the leading cause of transfusion-related mortality in the US, with an estimated mortality rate of 5% to 8% of transfusion-related deaths.^[21]^ Whereas, TACO is the second most common cause of transfusion-associated fatality in the US from 2014 to 2018.^[22]^ Because it tends to affect critically ill patients, it’s difficult to ascertain the true incidence of TACO. Nevertheless, a recent report estimates that approximately 6% of transfusions in critically ill patients may be associated with TACO^[23,24]^ and 1 per 25,000 platelet transfusions are attributed to septic transfusion reactions.^[25]^

So, ATR is one of the known forms of transfusion adverse effects linked with increased morbidity and mortality, with socioeconomic implications at either the individual or community level, resulting in a variety of clinical and/or social issues, including death.^[10,21]^ In broad terms, there is a deficit of concrete data on ATRs in Ethiopia, although the problem is widespread. Thus, analyzing the magnitude of ATR in terms of contributing factors among in-patients receiving blood or blood component therapy was the aim of this study at Jimma University Medical Center (JUMC), southwest Ethiopia, which is critical in reducing the occurrence of transfusion reactions and their complications.

## 2. Methods

This study was conducted at JUMC, located in Jimma City 352 km southwest of Addis Ababa. The daily transfusion utilization of the center is an average of 10 units. While all adult blood or blood component transfused voluntary in-patients were included, individuals unwilling to be part of, persons with mental illness, unconscious and critically ill, and receiving dialysis were excluded from the study. We collected pertinent socio-demographic information and previous medical conditions, including any former transfusions, gravidness, and miscarriages, using designed forms administered through interviews.

During the clinical infusion process, body temperature, blood pressure, pulse rate, and respiratory rate were measured 15 minutes after the commencement of the transfusion and then subsequently hourly to the end of the transfusion at the same time, patients were observed for features of transfusion reaction, which included signs of fever, chills/rigors, itching, urticaria, nausea, tachycardia, restlessness, vomiting, dyspnea, anxiety, and headache. The monitoring was continued 4 hourlies up to 24 hours after transfusion.

Data on the date of blood donation, blood group of donor and recipient, number of units transfused, and type of blood component were recorded.

The investigation done in our blood bank for transfusion-related reactions was as follows:

- All the documents are rechecked to identify any clerical errors.
- The volume of blood left and any abnormality are rechecked.
- Recipient blood was tested for hemoglobin after transfusion.
- Grouping and crossmatching are repeated on the posttransfusion sample. At the same time, a pretransfusion sample is rechecked for grouping and cross-matching.
- Posttransfusion urine sample is sent to test for hemoglobinuria.

Given that blood transfusion is only one aspect of patient management and necessitates a thorough analysis of individual patient needs, the Ethiopian National Guideline for Appropriate Clinical Use of Blood indicated that; whole blood transfusion is considered appropriate only in most cases of massive hemorrhage and exchange transfusion, PRBC for symptomatic anemic patients with a strict consideration of patients’ age (neonate, infant, child, adult), surgery (pre- and intra-op). Otherwise, plasma components and/or derivatives should be transfused to manage coagulation disorders and factor deficits.^[26]^

Data clearance, sorting, categorization, and organization were conducted before entering the data into SPSS^®^ statistical software version 20 (IBM Corps., Armonk, NY). This step ensures that the data are clean, well-organized, and ready for analysis. Summary expression of demographic and clinical features of study participants displayed by texts, tables, and figures. The multivariable binary logistic regression model was examined with reverse plausibility and progressive approach for parameters with a *P* value below .25 in the binary logistic regression. The mathematical validity of the final multifactorial logistic regression simulation was assessed using the Hosmer and Lemeshow test, and a final *P* value < .05 revealed the existence of a statistically significant relationship.

## 3. Results

### 3.1. Socio-demographic characteristics of study participants

A total of 384 participants were included in this study, with more than half of 214 (55.7%) of them being males. The age of the studied participants ranged from 18 to 85 years, and the median age of transfused patients was 35 with an interquartile range of (IQR ± 21) years (Table 1).

**Table 1:** Socio-demographic features of in-patients transfused with blood or blood products at JUMC from October 1 to December 30, 2020.

Character		Figure	Percentage (%)
Sex	Male	214	55.7%
	Female	170	44.3%
Age^a^	18 to 30	149	38.8%
	31 to 40	102	26.6%
	41 to 50	61	15.9%
	51 to 60	30	7.8%
	>60	42	10.9%

a The median age of patients who received transfusion therapy was 35 years.

### 3.2. Clinical characteristics of study participants

Of the study participants, 176 (45.8%) had a previous transfusion history, of which 66 (37.5%) had a previous transfusion history episode of 2. Among 170 female study participants, 100 (58.8%) had a pregnancy history, and 41 (24.1%) had a past miscarriage. The prevailing clinical indications leading to transfusion are situated in hematological disorders (36.2%), followed by other medical illnesses (18.5%) (Table 2).

**Table 2:** Clinical characteristics of in-patients transfused with blood or blood products at JUMC from October 1 to December 30, 2020.

Characteristics		Frequency N (%)
Transfusion history (N = 384)	Yes	176 (45.8%)
	No	208 (54.2%)
Transfusion history (176)	One	65 (36.9%)
	Two	66 (37.5%)
	Three and more	45 (25.6%)
Abortion history (N = 170)	Yes	41 (24.1%)
	No	129 (75.9%)
Frequency of abortions (N = 41)	One	39 (95.1%)
	Two	2 (4.9%)
Pregnancy history (N = 170)	Yes	100 (58.8%)
	No	70 (41.2%)
Gravidity (N = 100)	One	21 (21%)
	Two	19 (19%)
	Three and more	60 (60%)
Primary diagnosis (N = 384)	Chronic diseases^a^	57 (14.8%)
	GI bleeding	12 (3.1%)
	Hematological disorder	139 (36.2%)
	Obstetric complication	40 (10.4%)
	Other medical illnesses	71 (18.5%)
	Surgical cases	32 (8.4%)
	Trauma	33 (8.6%)

a Chronic diseases: Kidney diseases, liver diseases, tuberculosis, and HIV.

### 3.3. Blood group, blood component type, characteristics of transfused blood, and frequency of transfused patients

**Figure 1.**
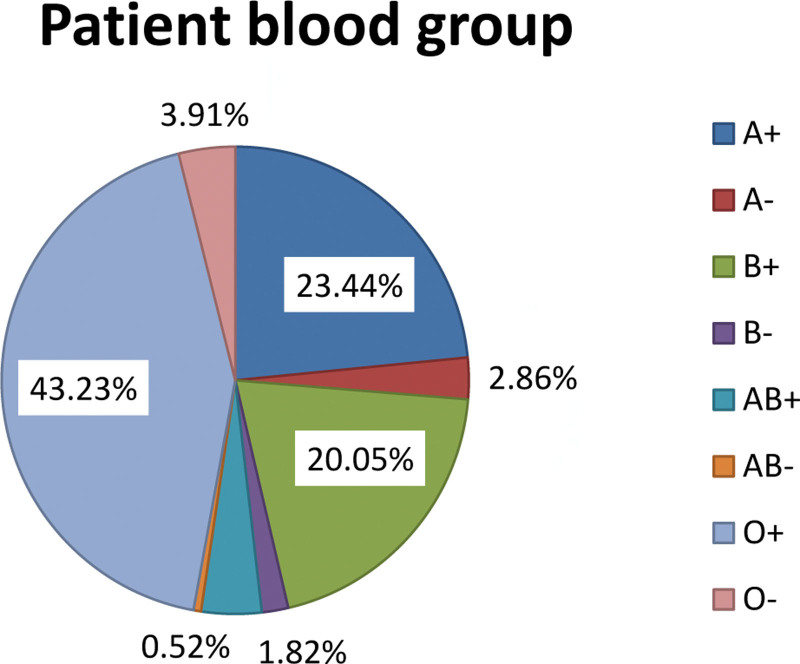
Blood group characteristics and frequency of adult transfused patients at JMC from October 1 to December 30, 2020.

Most patients 166 (43.23%) were transfused with O Rh D positive blood groups and AB Rh D negative were the less transfused blood group type 2 (0.52%) (Fig. 1).

Overall, 613 blood and blood product components were provided to 384 hospitalized patients, with an average of 1.60 units of either blood or blood product infused per person (1.25 for men and 2.03 for females). A large number of patients received whole blood transfers (nonleukocyte reduced) 363 (94.5%), followed by PRBCs (nonleukocyte reduced) 19 (4.9%) and platelet concentrates (made from whole blood) 2/384. Most patients received whole blood after a typical refrigerator retention time of 13 days (IQR ± 5), PRBC was 10 days (IQR ± 6), and platelet concentrate was 4 days. Two hundred thirty-eight (62%) patients got freshly drawn blood around 14 days, with a single unit of blood or blood component contributing to 63.8% of the clinical intervention (Table 3).

**Table 3:** Blood or blood product category and features of blood component related to patients received transfusion therapy at JUMC from October 1 to December 30, 2020.

Blood component features		Number (%)
Blood category	Whole blood	363 (94.5%)
	PRBC	19 (5%)
	PLT	2/384
Storage duration (refrigeration period)	Below 14 d	238 (62%)
	More than 14 d	146 (38%)
Units/Bags transfused	One unit	245 (63.8%)
	Two units	72 (18.8%)
	Three and more	67 (17.4%)
Total units transfused	613	

PRBC = packed red blood cells, PLT = platelet.

### 3.4. Transfusion reactions and associated factors

There were 22 ATR cases, accounting for 5.7% of the ATR incidence rate. Most of the reactions that occurred were FNHTR 14 (63.6%) followed by allergic reactions 8 (36.4%) with mild and moderate reactions (6/8) and (2/8), respectively (Table 4).

**Table 4:** Incidence and type of ATRs recorded among adult blood transfused in-patients at JUMC from October 1 to December 30, 2020.

Incidence of reaction and type		Frequency N (%)
Clinical occurrence	Reaction not reported	362 (94.3%)
	Reaction reported	22 (5.7%)
Reported types	Febrile nonhemolytic	14 (63.4%)
	Allergic	8 (36.4%)
	Mild allergic	6/8
	Moderate allergic	2/8

FNHTR = febrile nonhemolytic transfusion reaction.

**Figure 2.**
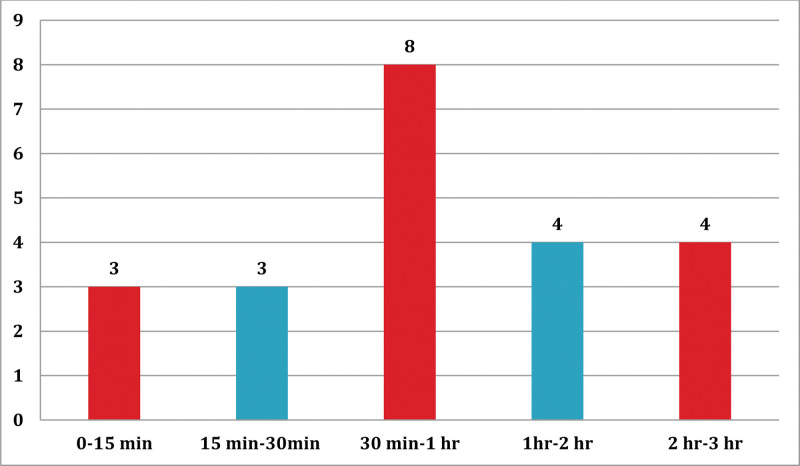
Onset of acute transfusion reaction after starting the transfusion among blood-transfused patients at JMC from October 1 to December 30, 2020.

Eight of the twenty-two (8/22) ATR cases occurred between 30 minutes to 1 hour after the start of the transfusion, while 14/22 ATR cases occurred within 1 hour of the initial transfusion. The maximum duration of developing the transfusion reaction was observed to be 2 to 3 hours during transfusion (Fig. 2).

**Figure 3.**
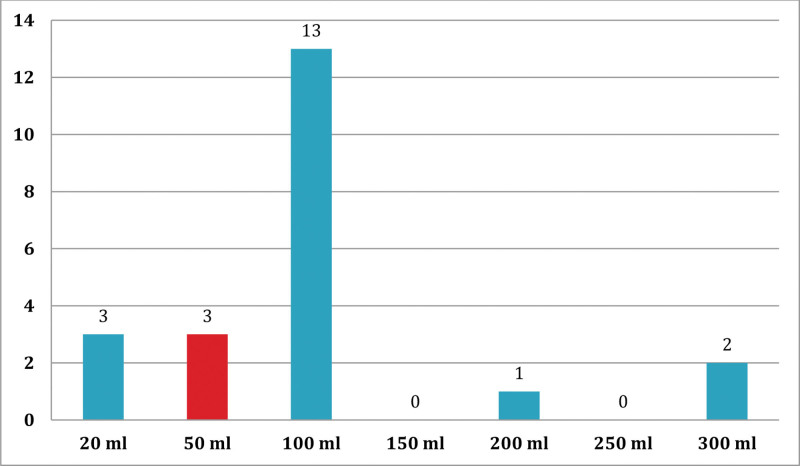
Amount of blood transfused before acute transfusion reaction among blood-transfused patients at JMC from October 1 to December 30, 2020.

Most of the reactions, (19 of the 22) cases occurred after transfusion of up to 100 mL of blood. Almost all reaction incidences were observed before the completion of the transfusion of the whole unit (Fig. 3).

ATR was more common in females (7.6% vs 4.2%), among people with previously received blood transfusion, (9.1% vs 2.9%), in patients with a past childbirth (9% vs 5.7%), miscarriage (17.1% vs 4.7%), in individuals who had been given more than 3 units (13.4% vs 4.1%), and in patients who had received relatively old blood (10.3% vs 2.9%). ATR was also detected in 90.9% of patients who received whole blood components. In the bivariate logistic regression analysis, ATR was associated with abortion history (COR = 4.2; 95% CI: 1.3–13.4), transfusion history (COR = 3.4; 95% CI: 1.3−8.8), transfusion ≥ 3 bags of blood (COR = 3.6; 95% CI: 1.5–8.9) and storage time (COR = 3.8; 95% CI: 1.5 − 9.5). In the multivariate analysis, transfusion history (adjusted odds ratio (AOR) = 3.3; 95% CI: 1.2−8.75, *P* = .018), storage time of transfused blood for more than 13 days (AOR = 3.85; 95% CI: 1.5–9.9, *P* = .005) and number of transfused units (≥3 units of blood/blood component) (AOR = 3.9; 95% CI: 1.52–9.8, *P* = .005) was substantially related with the development of ATR (Table 5).

**Table 5:** Bivariate and multivariate logistic regression analyses of associated factors of ATRs among adult transfused patients at JUMC from October 1 to December 30, 2020.

Variable		Reaction		COR* (95% CI)	AOR (95% CI)	*P* value
		Recorded	Not-developed			
Sex	Male	9 (4.2%)	205 (95.8%)	1	1	.377
	Female	13 (7.6%)	157 (92.4%)	1.9 (0.8–4.5)	1.6 (0.6–4.7)	
Storage duration	<14 d	7 (2.9%)	231 (97.1%)	1	1	.005
	≥14 d	15 (10.3%)	131 (89.7%)	3.8 (1.5–9.5)	3.85 (1.5–9.9)	
Transfused units	<3 units	13 (4.1%)	304 (95.9%)	1	1	.005
	≥3 units	9 (13.4%)	58 (86.6%)	3.6 (1.5–8.9)	3.9 (1.52–9.8)	
Transfusion history	No	6 (2.9%)	202 (97.1%)	1	1	.018
	Yes	16 (9.1%)	160 (90.9%)	3.4 (1.3–8.8)	3.3 (1.2–8.75)	
Abortion history	No	6 (4.7%)	123 (95.3%)	1		.015*
	Yes	7 (17.1%)	34 (82.9%)	4.2 (1.3–13.4)		

AOR = adjusted odds ratio, ATR = acute transfusion reaction, COR = crude odds ratio.

* *P* value for multivariate logistic regression.

## 4. Discussion

A transfusion reaction is generally defined as any adverse reaction that occurs in a patient during or after the administration of blood and/or blood products, ranging from mild symptoms to a life-threatening clinical condition. Acute transfusion reactions (ATRs) can cause serious adverse events and their incidence varies worldwide.^[27]^

The incidence of reaction among adult blood and blood product transfused patients in this study was found to be 5.7% (CI 3–8%). This result aligns with previous studies conducted in Japan (5.05%), Belgaum, India (4.41%), Nigeria (3.6%), and Ethiopia (5.2%).^[10, 28–30]^ However, it is lower than what was reported in studies conducted in Israel (11%) and Nigeria (26.3%).^[31, 32]^ The Israeli study focused on elderly patients with a median age of 82 ± 9 which may explain the higher incidence observed. However, the participants in our study had a median age of 35 years old, with only a small proportion being older age (14.8%). The Nigeria study focused solely on pregnant ladies, especially those who had partial abortions, and placenta cover-up and fetal-maternal bleeding (*placenta previa* and *fetal-maternal hemorrhage*), which could contribute to the occurrence of ATR.^[31]^ Multigravida, in particular, mainly results from the production of antibodies to leukocytes or PLT antigens due to the transfer of fetal blood cells to maternal circulation during pregnancy or at the time of delivery at large. This suggests that mothers having preformed white cell antibodies after conception or miscarriage are prone to develop FNHTR and hypersensitivity responses, as they receive blood components with leukocytes.^[4]^

However, the current investigation revealed an increased ATR rate than the research executed from Japan (2.6%).^[33]^ Leukocyte-reduced blood components were employed in the Japan trial, but this was not a frequent practice in our facility, so nonleukocyte-reduced components were primarily used. FNHTR, a prevalent kind of ATR in nonleukocyte-reduced blood, is typically triggered by an immunological response that includes antileukocyte antibodies.^[34]^ Transfusing leukocyte-depleted blood can decrease the likelihood of FNHTR, HLA alloimmunization, and PLT refractoriness. However, it may not necessarily lessen the incidence of other types of ATRs.^[35]^

In this study, FNHTR (63.6%) and allergic (36.4%) were the only observed types of transfusion-related reactions. The current finding is consistent with research undertaken in the US (61.4% and 35.7%), India (60.4% and 31.2%), Saudi Arabia (41.9% and 34.4%), Nigeria (47.7% and 24.5%), and Zimbabwe (58.5% and 31.6%).^[31, 36–39]^ These studies revealed that FNHTR was the most commonly observed type of transfusion reaction. However, contrary to this study’s findings, studies conducted in Japan, Malaysia, and Ethiopia^[10, 33, 40]^ showed a higher frequency of allergic reactions. Two cases of moderate allergic reaction were observed in this study. Such reactions are mainly caused by soluble substances, typically proteins (albumin, complement component, IgG, and IgA) found in the donor’s plasma,^[17]^ leading to the activation of mast cells due to antigen interactions with preformed IgE antibodies resulting in the release of bioactive mediators.^[41]^ The specific causes of these reactions were not determined in our study, and there were no indications of mismatch before infusion with negative DAT results. However, these patients presented indications of hypersensitive reactions; for instance, “*wheezing,”* “*itching,”* “*urticaria,” and* “*facial and body flushing.” The transfusion was promptly halted, and standard treatment was administered.*

No cases of AHR, TRALI, and TACO were recorded in this research. This could be evidenced by the reduced number of individuals included in this study related to reports from elsewhere. McDonald et al reported a 0.03% rate of confirmed cases out of 1239,029 platelet components, whereas Li et al found that 6% of transfused patients developed TACO out of 901 individuals, and Rana and colleagues reported 17 cases of possible TRALI and 25 cases of TACO out of 8902 bags of blood transfusions.^[19, 23, 42]^ It is also important to consider that there might be underreporting of bacterial contamination cases since microbiological investigations were not performed in most suspected cases.

This study also documented that the majority of the transfusion reactions occurred between 30 minutes and 1hr after the start of the blood transfusion. However, a study conducted in India reported that most reactions occurred within 15 minutes of starting the transfusion.^[8]^ The difference in timing might be due to the types of ATRs observed. The above-mentioned study reported more severe ATRs, while in this study most of the reactions observed were mild.

Acute transfusion reaction was 3.85 times more likely to occur among patients transfused with blood that has been kept for a long time than among patients transfused with short-stored blood. Studies conducted in Nigeria and Ethiopia^[10, 31]^ have reported similar findings. This is due to the increased bio-chemicals and immunological changes that affect blood cell viability and the recipient’s response to transfusion. Moreover, the risk of acute reactions, especially in febrile nonhemolytic and sensitivity reactions increases as the length of storage increases and corresponds to the exponential rise in cytokine concentration observed with storage time.^[41]^ Still, blood or components that remained kept for longer periods are linked with transfusion reaction but do not necessarily cause critical negative health impacts.^[10]^

US and Ethiopia study reports^[10, 34]^ evidenced that, there was a direct and statistically significant relationship between how many units of blood were administered with the clinical occurrence of ATRs. A single unit increase in transfusion for patients who had received multiple units of blood raised the risk of ATR by 3.9 folds. This might be due to repeated transfusion can result in the production of alloantibodies against blood cell antigens, which complicates the subsequent transfusions because in multiple transfusions biochemical, cytokines, and antibodies complexity happen leading to ATRs.^[43]^

In this study, a statistically weighty connection was found between ATR and former blood or component infusions, which is similar to earlier reports from the USA, Nigeria, and Ethiopia.^[10, 28, 34]^ The possible reason might be due to the immune system sensitization and production of antibodies to certain RBC, WBC, and PLT antigens after previous transfusion. This sensitization may cause the development of ATRs in a subsequent blood transfusion.^[44]^ Women who had a history of previous miscarriage were more prone to develop ATRs than those without those incidences. The rationale is that, during an abortion incidence or process, fetal antigens may enter maternal circulation, which leads to sensitization. When transfusion takes place, the immune system produces antibodies that cause ATRs.^[45]^

### 4.1. Strengths and limitations of the study

The strength of this study was that trained physicians with knowledge of ATRs individually evaluated each adverse transfusion event using predefined, internationally accepted ATRs criteria, and also evaluated any transfusions administered at night or on weekends. The limitations of this study were the short study period and the small sample size. In addition, blood cultures were not performed as a differential diagnosis. This study did not look into the relationship between the independent variables and ATR variants because the number of study participants who developed the clinical outcome of interest, ATR, was minimal (n = 22) for several variables.

### 4.2. Conclusion and recommendation

Adverse reaction following blood transfusion is a common complication that should be kept in mind and blood transfusion should be given when necessary. The most common transfusion reaction reported was a febrile incidence followed by an allergic one. The majority of the reactions were observed in patients with a clinical history of prior transfusion, including the number of units administered, a history of miscarriage, and blood component length of storage. Patients with a previous history of transfusion and more than 2 units transfused are more likely to develop ATR. This can be decreased by administering leukocyte-reduced blood products and maintaining ongoing clinical monitoring.

Hemovigilance is necessary in this situation; any ATR clinical interactions should be reported to the blood bank and transfusion committee. The hemovigilance system must be well-coordinated among the blood transfusion service, hospital clinical staff and transfusion laboratories, hospital transfusion committee, regulatory agency, and national health authority. As a result, building a hemovigilance system to monitor, collect, and analyze data on the adverse effects of blood transfusions on a local and national scale will lower the occurrence of acute transfusion responses.

## Acknowledgments

The authors thank all staff of JUMC and study participants for their important contributions and participation.

## Author contributions

**Conceptualization:** Edosa Tadasa, Wondimagegn Adissu, Lealem Gedefaw.

**Data curation:** Edosa Tadasa, Wondimagegn Adissu, Lealem Gedefaw.

**Formal analysis:** Edosa Tadasa, Wondimagegn Adissu, Lealem Gedefaw.

**Investigation:** Edosa Tadasa, Wondimagegn Adissu, Lealem Gedefaw.

**Methodology:** Edosa Tadasa, Wondimagegn Adissu, Misgana Bekele, Gebeyaw Arega, Lealem Gedefaw.

**Project administration:** Edosa Tadasa, Wondimagegn Adissu, Lealem Gedefaw.

**Resources:** Edosa Tadasa, Wondimagegn Adissu, Lealem Gedefaw.

**Software:** Edosa Tadasa, Wondimagegn Adissu, Lealem Gedefaw.

**Supervision:** Edosa Tadasa, Wondimagegn Adissu, Lealem Gedefaw.

**Validation:** Edosa Tadasa, Wondimagegn Adissu, Lealem Gedefaw.

**Visualization:** Edosa Tadasa, Wondimagegn Adissu, Lealem Gedefaw.

**Writing – original draft:** Edosa Tadasa, Misgana Bekele, Gebeyaw Arega.

**Writing – review & editing:** Edosa Tadasa, Wondimagegn Adissu, Misgana Bekele, Gebeyaw Arega, Lealem Gedefaw.
